# Engineering a Humanised Niche to Support Human Haematopoiesis in Mice: Novel Opportunities in Modelling Cancer

**DOI:** 10.3390/cancers12082205

**Published:** 2020-08-06

**Authors:** Alvaro Sanchez-Herrero, Isabel A. Calvo, Maria Flandes-Iparraguirre, Marietta Landgraf, Christoph A. Lahr, Abbas Shafiee, Froilán Granero-Molto, Borja Saez, Manuel M. Mazo, Bruno Paiva, Elena de Juan Pardo, Andrew Nicol, Felipe Prosper, Laura J. Bray, Jacqui A. McGovern

**Affiliations:** 1 Institute of Health and Biomedical Innovation, Queensland University of Technology (QUT), Kelvin Grove, Queensland 4059, Australia; a2.sanchez@qut.edu.au (A.S.-H.); Marietta.landgraf@gmx.de (M.L.); christoph.lahr@googlemail.com (C.A.L.); a.shafiee@uq.edu.au (A.S.); 2 Centro de Investigación Médica Aplicada (CIMA), IDISNA-Instituto de Investigación Biosanitaria de Navarra, 31008 Pamplona, Spain; icalvoa@unav.es (I.A.C.); mariaflandes@hotmail.com (M.F.-I.); fgranero@unav.es (F.G.-M.); bsaezoch@unav.es (B.S.); mmazoveg@unav.es (M.M.M.); Bpaiva@unav.es (B.P.); fprosper@unav.es (F.P.); 3 Hematology-Oncology, Center for Applied Medical Research (CIMA), University of Navarra, 31008 Pamplona, Spain; 4 Department of Hematology, Clínica Universidad de Navarra, 31008 Pamplona, Spain; 5 Cell Therapy Program, Center for Applied Medical Research (CIMA), University of Navarra, 31008 Pamplona, Spain; 6 Herston Biofabrication Institute, Metro North Hospital and Health Service, Brisbane 4029, Australia; 7 Translational 3d Printing Laboratory for Advanced Tissue Engineering (T3mPLATE), Harry Perkins Institute of Medical Research, QEII Medical Centre, Nedlands and Centre for Medical Research, The University of Western Australia, Perth 6009, Australia; elena.juanpardo@uwa.edu.au; 8 School of Engineering, The University of Western Australia, Perth 6009, Australia; 9 School of Mechanical, Medical and Process Engineering, Science and Engineering Faculty, Queensland University of Technology (QUT), Brisbane 4000, Queensland, Australia; 10 University of Queensland, Greenslopes Private Hospital, Brisbane, QLD 4120, Australia; anic9909@myhaematologist.com.au

**Keywords:** humanised mouse model, bone marrow, tumour microenvironment, CD34+ haematopoietic stem cell, tissue engineering, multiple myeloma

## Abstract

Despite the bone marrow microenvironment being widely recognised as a key player in cancer research, the current animal models that represent a human haematopoietic system lack the contribution of the humanised marrow microenvironment. Here we describe a murine model that relies on the combination of an orthotopic humanised tissue-engineered bone construct (ohTEBC) with patient-specific bone marrow (BM) cells to create a humanised bone marrow (hBM) niche capable of supporting the engraftment of human haematopoietic cells. Results showed that this model supports the engraftment of human CD34^+^ cells from a healthy BM with human haematopoietic cells migrating into the mouse BM, human BM compartment, spleen and peripheral blood. We compared these results with the engraftment capacity of human CD34^+^ cells obtained from patients with multiple myeloma (MM). We demonstrated that CD34^+^ cells derived from a diseased BM had a reduced engraftment potential compared to healthy patients and that a higher cell dose is required to achieve engraftment of human haematopoietic cells in peripheral blood. Finally, we observed that hematopoietic cells obtained from the mobilised peripheral blood of patients yields a higher number of CD34^+^, overcoming this problem. In conclusion, this humanised mouse model has potential as a unique and patient-specific pre-clinical platform for the study of tumour–microenvironment interactions, including human bone and haematopoietic cells, and could, in the future, serve as a drug testing platform.

## 1. Introduction

Over 80% of drugs fail to obtain market approval, despite showing promising results in pre-clinical studies. This is mainly due to the lack of clinically predictable animal models that faithfully represent human disease [1], particularly in the field of oncology [2]. Pre-clinical studies are hampered by the biological differences between human and murine physiology in terms of regulatory proteins and cellular dynamics [2,3,4]. In particular, the role of the immune system may be obscured if experiments are conducted within a murine immune system context [5], which has been proven to play a key role in cancer disease [6].

Humanised mice emerged in an effort to overcome species differences, with animals featuring functional human cells and tissues [7]. One of the aspects that can be humanised is the haematopoietic system [8] and it has been recognised as a promising tool for unravelling the complex mechanisms that exist between the immune system and tumour cells. In the 1990s it was first reported that peripheral blood mononuclear cells (Hu-PBL-*Scid*) [9] and haematopoietic stem cells (HSCs) (Hu-SRC-*Scid*) [10] were able to engraft in immunodeficient mice and generate human haematopoiesis. This revolution in pre-clinical research was facilitated by the development of immunodeficient mice, especially the NOD/SCID gamma (NSG) strain in the early 2000s allowing the development of xenograft models [11]. This has been extensively studied and numerous groups have succeeded in creating humanised haematopoiesis in mice [7].

Currently, the gold standard in humanising the immune system of mice involves the implantation of cord blood-derived CD34^+^ cells into previously gamma-irradiated NSG mice [12], which is the most accepted mouse strain for xenotransplantation and tumour modelling. However, other sources of CD34^+^ cells, such as the bone marrow (BM) [13] and peripheral blood mononuclear cells [14], can also be employed, leading to the reconstitution of the haematopoietic system. This offers the opportunity of generating a patient-specific haematopoietic system in the mice, by engrafting patient-derived CD34^+^ cells, something that cannot be achieved with cord blood derived CD34^+^ cells. This would be especially relevant for those conditions in which the immune system has anomalies as this ‘diseased’ immune system could also be modelled in the mouse.

In addition to the haematopoietic system, the BM microenvironment also plays a key role in cancer progression, including the maintenance of the cancer stem cells [15]. Numerous cancer research groups have succeeded in partially humanising the BM microenvironment, such as in multiple myeloma (MM) [16] and also in solid tumours, such as osteosarcoma [17], prostate cancer bone metastasis [18] and others. These models allow the study of interactions between cancer cells and the tumour microenvironment, such as with bone cells [19]. Furthermore, humanisation is not only relevant to representing microenvironment interactions, but is also a requirement for testing certain drugs. For instance, the model is used for testing monoclonal antibodies (mAb) that do not show cross-reactivity with murine tissue [20]. This is especially relevant if the therapeutic strategy is directed towards the microenvironment, for instance, bone anti-resorptive therapy in MM [21] or the use of an anti-hypoxic therapy that prevents growth of myeloma cells [22].

We have come to a point where animal models are becoming increasingly complex; thus, incorporating novel technologies that offer an innovative set of strategies such as tissue engineering and regenerative medicine seems to be a necessary step to improve the current landscape of mouse modelling [23]. In our group we have recently established a humanised mouse model by engineering isolated humanised niches. This model results in the formation of a bone organ with both human cellular and extracellular components capable of supporting the engraftment of various cancer cell types such as breast and prostate cancer [4,24,25]. Following this approach, engraftment of human haematopoietic cells in a humanised tissue-engineered bone construct (hTEBC) implanted orthotopically around the femur of NSG mice was generated [26]. However, despite these significant advances in the establishment of humanised mice models, there is still a lack of patient-specific tools to investigate the role of the immune system in healthy and malignant haematopoiesis, including cancer. Moreover, current models fall short of integrating the complexity of the human bone microenvironment, which is crucial in order to study tumours with bone and/or marrow tropism, such as prostate cancer or MM.

Here, we present the first steps towards the development of a fully patient-specific mouse model containing a humanised and patient-specific haematopoietic niche in the mouse that supports the engraftment of patient-derived BM CD34^+^ cells. This is demonstrated using haematopoietic cells from non-tumour and MM patients as examples of diseased marrow. This work sets a basis towards establishing fully patient-specific and clinically relevant humanised mouse models to enable efficient drug testing and the investigation of immunological interactions for various diseases.

## 2. Results

### 2.1. The Orthotopic Humanised Tissue Engineered Bone Construct (ohTEBC) Forms a Humanised Bone Marrow Niche In Vivo

**Figure 1:**
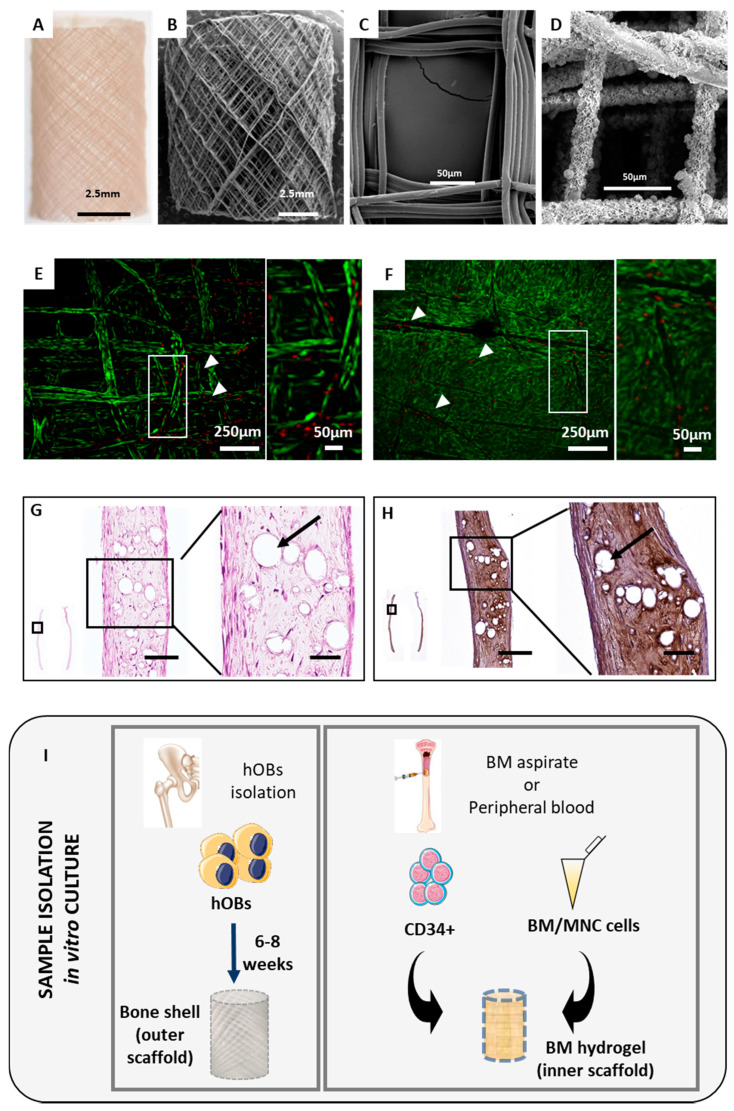
Melt electrowritten (MEW) tubular mPCL scaffolds support human osteoblast (hOB) growth and are suitable for in vivo implantation. Orthotopic tubular PCL scaffolds were imaged with a stereomicroscope (**A**). MEW tubular scaffolds were printed, and pore size and fibre diameter were measured using scanning electron microscopy (SEM) images, obtaining an average of 156.5 µm and 7.7 µm, respectively (**B**,**C**). After CaP coating, SEM images were also acquired to verify the presence and homogenous distribution of the coating (**D**). FDA/PI staining showed high viability of the cells 1 week after hOB seeding (**E**) and 8 weeks after hOB seeding (**F**) on the tubular PCL scaffold, as revealed by the high number of living cells (stained in green), compared to the low number of dead cells (stained in red, with white arrowheads indicating areas of the scaffold with some dead cells and a zoomed-in area). Tubular scaffolds were cut longitudinally into 5 µm sections to evaluate the ability of hOBs cultured in tubular PCL scaffolds to produce an extracellular matrix ECM. H&E staining (**G**) showed the morphology and distribution of the cells within the scaffold. Human-specific Col1 immunohistochemistry (IHC) was used to verify the human origin of the matrix (**H**). Black arrows indicate the location of PCL scaffold fibres. Scale bars: 100 µm, 50 µm. The implant consisted of two concentric MEW scaffolds; the outer scaffold had pre-cultured hOBs that formed the human bone cortical shell, and the inner scaffold formed the bone marrow (BM) compartment by incorporating fibrin glue containing the hBM and CD34^+^ cells isolated from either a BM aspirate or peripheral blood. The hOBs were isolated from bone chips obtained from donors following hip replacement surgery (**I**).

For the establishment of the orthotopic humanised tissue-engineered bone construct (ohTEBC), tubular medical-grade polycaprolactone (mPCL) scaffolds were printed in a custom made melt electrowritten (MEW) device, as described previously [27] (Figure 1A–C). Scaffolds were then calcium phosphate (CaP)-coated (Figure 1D) and seeded with primary human osteoblasts (hOBs) isolated from hip or knee human bone chips (Figure 1I). The viability of hOBs in the scaffold was confirmed using fluorescein diacetate (FDA) and propidium iodide (PI) staining to detect living and dead cells, respectively (Figure 1E,F). Within the first week of culture, hOBs were only covering the scaffold fibres (Figure 1E). After eight weeks, the scaffold was completely confluent, with the hOBs bridging the gaps between the pores (Figure 1F). After reaching confluency, the culture was switched to osteogenic media to stimulate the hOBs to secrete a mineralised extracellular matrix (ECM).

To characterise the morphology of the hOBs cultured in osteogenic media and the ECM production in vitro, histological analysis was conducted. Haematoxylin and eosin (H&E) staining was performed on 5-µm-thick longitudinal sections of the cultured scaffolds. From the H&E staining (Figure 1G) it was confirmed that the hOBs were able to infiltrate throughout the entire scaffold, secreting a rich ECM composed mainly of human type 1 collagen (Col1), which is characteristic of bone tissues [28] (Figure 1H).

Following confirmation of viable hOBs covering the tubular scaffolds and depositing ECM, BM cells were isolated from the BM or peripheral blood of patients after red blood cell lysis. CD34^+^ cells were also obtained from the same sample by using magnetic beads (Figure 1I).

**Figure 2:**
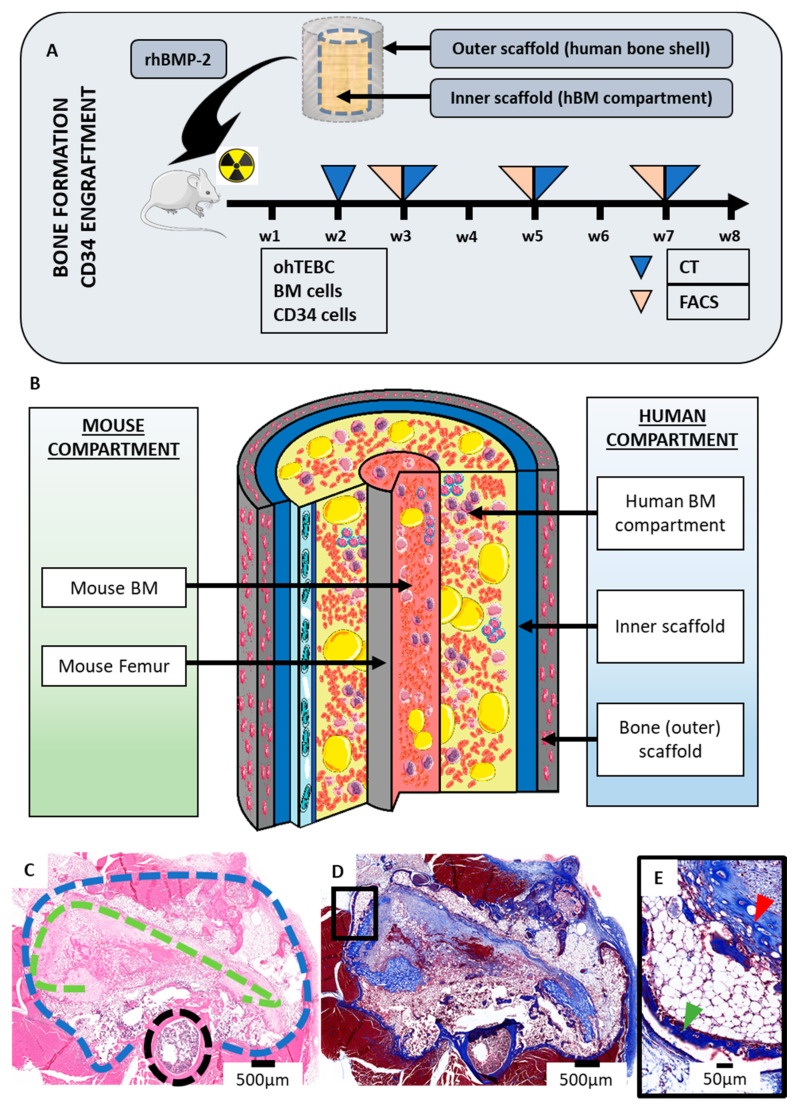
The establishment of the ohTEBC results in the formation of a bone organ within the mouse. The double coaxial scaffold construct was implanted orthotopically with rhBMP-2 around the femur of previously irradiated NSG mice. Bone formation was monitored by in vivo CT scans and engraftment of the CD34^+^ cells was measured by flow cytometry every 2–3 weeks (**A**). After in vivo implantation, the construct contained an hBM compartment within the bone cortical shell formed by the scaffold containing the pre-cultured hOBs (**B**). Representative histological cross sections of a construct. The H&E staining (**C**) allows differentiation between the outer bone scaffold (blue dashed line), the inner scaffold that formed the hBM compartment (green dashed line) and the murine femur (black dashed line). Masson’s trichrome staining (**D**) demonstrates deposition of the ECM in the construct, depicting collagen in blue. Zoomed-in detail of an area of Masson’s trichrome staining showing the outer and inner scaffolds, indicated by the green and red arrowheads, respectively (**E**).

To generate the humanised BM microenvironment, scaffolds were implanted orthotopically around the right femur of previously irradiated six–eight-week-old NSG mice. Animals were then implanted with the ohTEBC, composed of two concentric tubular mPCL MEW scaffolds. The outer scaffold contained the hOBs, and the smaller inner scaffold incorporated a fibrin glue hydrogel containing the BM enriched with CD34^+^ cells. Bone morphogenic protein 2 (rhBMP-2) was added to the interface of the scaffolds to stimulate mineralisation of a humanised bone shell around the BM (Figure 2A). A 0.5 mm cortical window was created in the mouse femur to allow communication between the murine BM and the ohTEBC. After in vivo implantation, the ohTEBC formed a clearly differentiated humanised BM compartment (Figure 2B), as shown by H&E staining (Figure 2C), where the bone (outer) scaffold formed an organised cortical shell comprising the newly formed humanised compartment. Masson’s trichrome staining showed large collagen deposition in the newly formed compartment, allowing clear differentiation between a densely packed outer scaffold and the inner scaffold, which contained the implanted hBM cells (Figure 2C,D). In these studies, samples from aged healthy donors were used (70 years old), resulting in the observation of a high infiltration of adipocytes (Figure 2D,E), characteristic of elderly hBM [29]. The presence of adipocytes in the model is a relevant feature, as it has been proven that marrow adipocytes contribute to bone disease [30] and tumour growth [31] in MM patients.

### 2.2. The ohTEBC Is Able to Support Human Haematopoiesis

**Figure 3:**
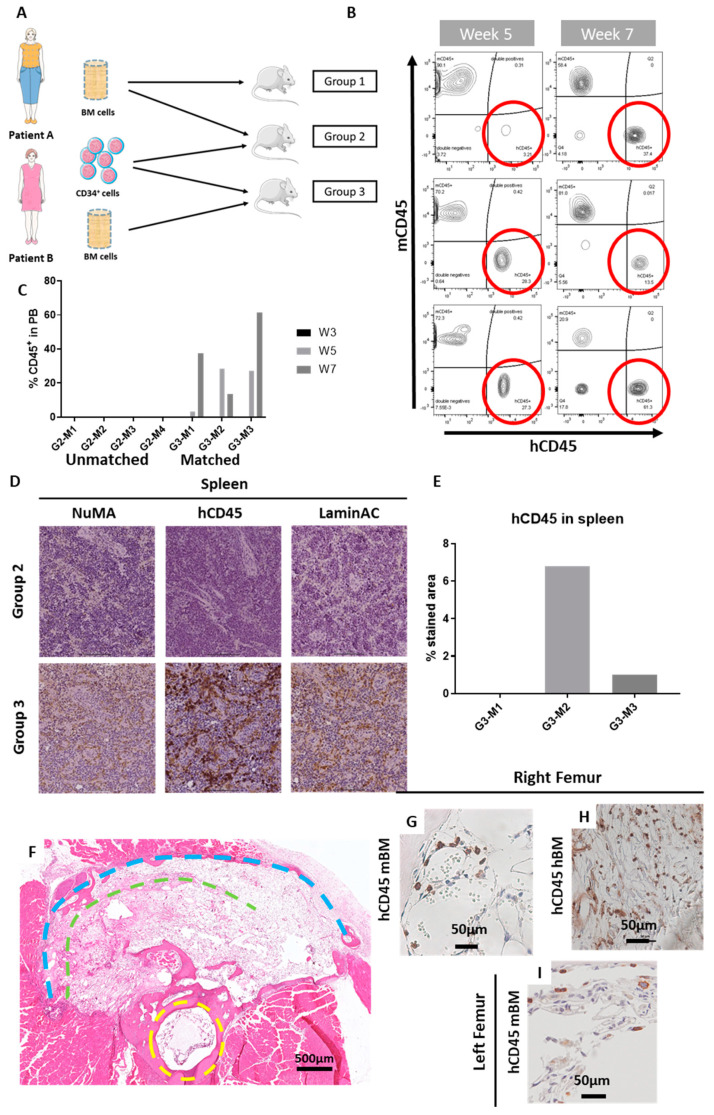
CD34^+^ isolated from aged patients were able to establish a human haematopoietic system in the mice. Overview of the in vivo study. Group 1 (*n* = 4 mice) did not receive any CD34^+^ cells, whereas Groups 2 (G2) and 3 (G3) were implanted with 85,000 CD34^+^ cells isolated from Patient B. Group 3 (*n* = 3 mice; G3-M1, G3-M2, G3-M3) had matched cells from the same patient, and Group 2 (*n* = 4 mice; G2-M1, G2-M2, G2-M3, G1-M4) had cells from different patients (**A**). Flow cytometry was utilised to monitor cell engraftment. After 5 weeks (**B**), hCD45^+^ cells were detectable in peripheral blood of the mice, with values ranging from 3.21% to 27.3%. Flow cytometry at week 7 verified their engraftment and was increased in some cases (**C**). Immunohistochemical analysis revealed the presence of hCD45^+^ cells in the spleen of the mice that receives the BM and CD34^+^ cells from the same patient (**D**), with up to 6% of hCD45^+^ stained area (**E**). H&E staining of a cross section of a mouse leg, with the yellow dashed line indicating the mouse femur and the green and blue representing the inner and outer implanted scaffolds, respectively (**F**). Further immunohistochemical analysis assisted to locate hCD45^+^ cells in the right femur of the mice (**G**–**H**), in both the murine BM compartment (**G**) and in the hBM compartment (**H**). hCD45^+^ cells also migrated to the contralateral, non-operated leg (**I**).

Once the ohTEBC was established, it needed to be determined whether this construct could support long-term engraftment of human CD34^+^ cells. First, a pilot experiment with samples from healthy BM was designed. We hypothesised that the implantation of hCD34^+^ cells, along with hBM mononuclear cells, would facilitate haematopoietic cell engraftment, as it provides a patient-specific microenvironment, and hence would enable the reconstitution of a humanised haematopoietic system in NSG mice. We designed three different groups, using two different patients of similar age and gender (70-year-old female patients) implanted in a total of 12 mice. Aged patients were utilised in this study because this system is intended to model conditions of older adults, such as multiple myeloma, with a third of patients being over 75 years old [32], or bone metastasis in breast or prostate cancer, both of which predominantly affect older adults [33]. BM cells from Patient A were implanted in the animals of Groups 1 and 2, and BM cells from Patient B were implanted in the animals of Group 3 (Figure 3A). To assess the ability of the CD34^+^ cells to engraft, CD34^+^ cells from Patient B were implanted in the construct of animals from Groups 2 and 3. Hence, we had a control group with no CD34^+^ cells, Group 2 with unmatched patients for the BM cells and the CD34^+^ and Group 3 with matched patients (BM cells and CD34^+^ cells were isolated from the same patient, Figure 3A). We hypothesised that engraftment would be superior when the BM and the CD34^+^ cells were from the same patient.

To monitor engraftment of haematopoietic cells, peripheral blood was obtained via retro-orbital bleeding and was analysed at weeks 3, 5 and 7. The frequency of human CD45^+^ (hCD45^+^) cells in peripheral blood was measured as an indication of successful engraftment. At week 3, no human cells were detected in the peripheral blood of any of the animals. Starting at week 5, hCD45^+^ cells could be found in peripheral blood, suggesting that the CD34^+^ cells had engrafted in the construct and were repopulating the haematopoietic system. Interestingly, the cells only engrafted in Group 3, in which the BM and the CD34^+^ cells were from the same patient (Figure 3B,C).

In mice, the spleen acts as a haematopoietic organ [34]; hence, the detection of migration of hCD45^+^ cells to this organ is a good demonstration of a successful engraftment of the CD34^+^ cells in the model. Consequently, to further elucidate the potential CD34^+^ cell engraftment, spleen samples from the three different groups were fixed, sectioned and stained for hCD45^+^.

Importantly, we only found infiltration of human cells within the spleens from Group 3 (which received the BM and CD34^+^ cells from the same patients). To further verify the human origin of these cells, human specific antibodies raised against the nuclear mitotic apparatus (NuMA) and LaminA/C proteins were employed and were found to be positive in the same areas as the hCD45^+^ staining (Figure 3D).

In addition to the spleen, we performed histological analysis on the right femur, containing the ohTEBC, and the contralateral, non-operated left leg. Positive hCD45 cells were found in the human BM compartment, where the cells were initially implanted. Interestingly, hCD45^+^ cells were also found in the murine BM of both the operated and the non-operated leg, indicating that these cells were fully engrafted in the mouse, as they were homing to the different haematopoietic organs after eight weeks (Figure 3D–I).

**Figure 4:**
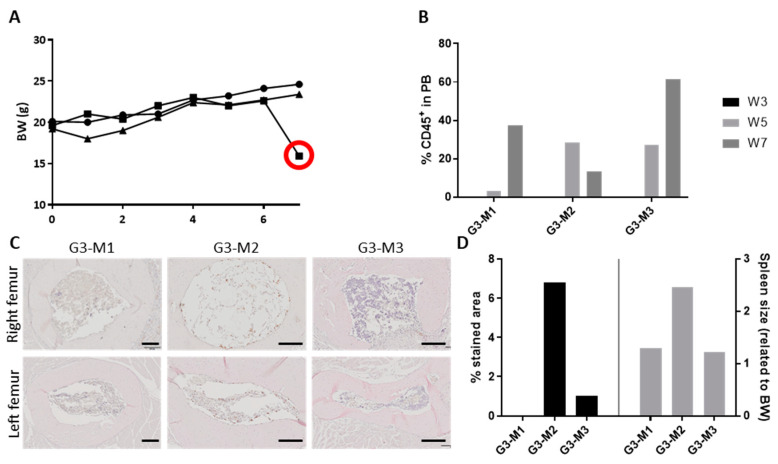
Excessive engraftment of CD34^+^ cells might cause graft vs. host disease (GvHD)-like symptoms. One of the mice showed signs of GvHD. First, a weight loss of 20% in less than a week (**A**). In addition, a decrease in the number of hCD45^+^ in peripheral blood (**B**) and reduced haematopoietic tissue in the BM, as compared with other mice (**C**). The spleen size was enlarged, and more hCD45^+^ infiltration occurred, which could have affected the spleen tissue (**D**). Scale bars: 200 µm. BW: body weight, G3-M1: group 3, mouse 1.

High levels of human cell engraftment could potentially react against murine tissues. One of the mice in the study showed some reminiscent signs of graft vs. host disease (GvHD), including rapid weight loss and a 50% reduction in the circulating hCD45^+^ cells (Figure 4A,B). Furthermore, histological analysis revealed a lower density of haematopoietic tissue in the BM (Figure 4C). Moreover, the mouse that showed GvHD signs had the highest infiltration of hCD45^+^ cells in the spleen (6.8%) (Figure 3E) and a splenomegaly with a spleen double the size (spleen weight related to body weight) than the other animals (Figure 4D). NSG mice are more prone to the development of GvHD signs, as the reconstitution levels of human cells tend to be higher than in other strains [35]. Although the signs indicated GvHD disease, this could not be confirmed.

### 2.3. CD34^+^ Cell Implantation Does Not Significantly Affect Bone Formation

We have demonstrated that engineering a humanised BM orthotopically facilitates the engraftment of the CD34^+^ cells. This construct also forms a humanised bone cortical shell around the mouse femur, as demonstrated here and in previous studies with the ohTEBC [26]. The presence of a humanised haematopoietic system might affect the bone formation rate, as the CD34^+^ cells are implanted together with the scaffold containing hOBs We intended for this model to allow the study of interactions between the tumour and the bone cells, as bone is a frequent site of metastasis for a number of cancers [36], and in some cases, tumour cells directly interact with the bone cells. One such example of this is MM, where tumour cells create a positive feedback loop in the bone [37,38].

**Figure 5:**
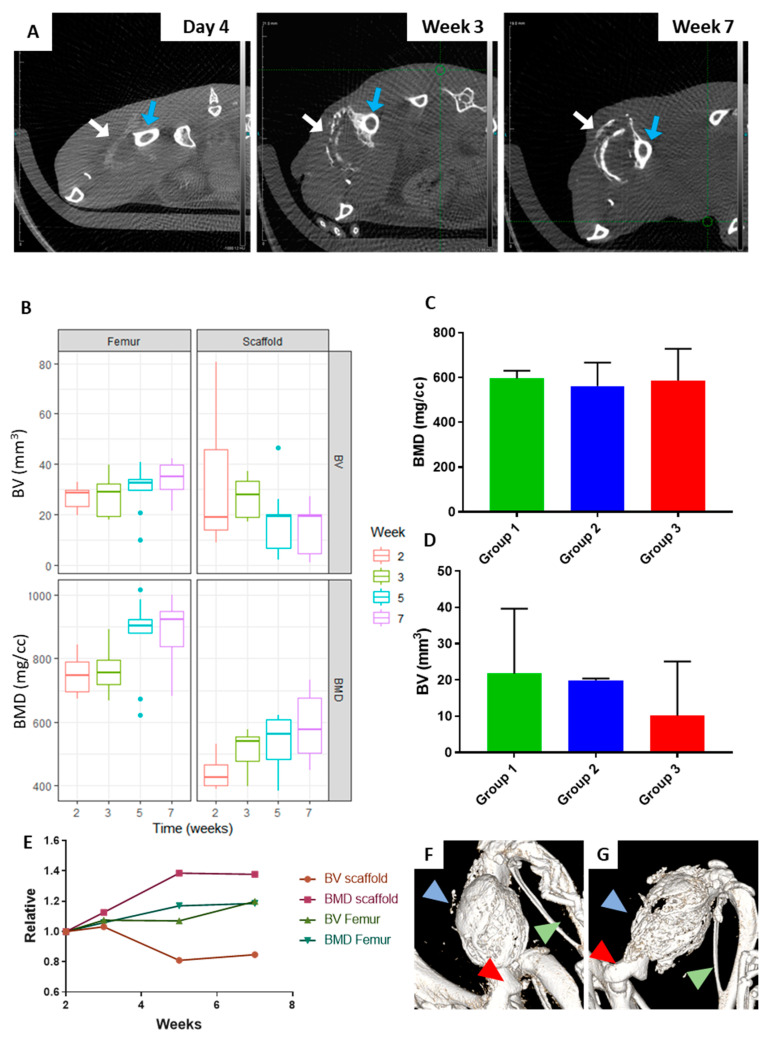
CD34^+^ cell implantation does not affect the bone formation rates in vivo. (**A**) Analysis of the bone formation over a period of 8 weeks from in vivo µCT (bone volume (BV) and bone mineral density (BMD)) depicting a transversal plane of a representative mouse. Blue arrows indicate the mouse femur and white arrows indicate the newly formed bone. (**B**) The bone volume (BV) of the scaffold reached a peak on week 2 and stabilised after week 4, indicating the formation of an organised cortical shell. After this time, the BV did not significantly change. On the other hand, the bone mineral density (BMD) of the scaffold increases with time, corroborating the organisation and growth of the bone organ. The BMD of the scaffold increased over time. The BV and BMD of the mouse femur were stable and tended to increase slightly, as per normal body growth. Lines in boxplot indicate the median, top and bottom lines corresponding to the 25% and 75% percentiles. The dots indicate outliers. (**C**) Average values from the 3 different groups at week 7: control (group 1), unmatched (group 2) and matched (group 3), showing no statistical difference in the scaffold BMD (**C**) or the BV (**D**). (**E**) Representative example of an individual mouse, showing the stabilisation of the scaffold’s BV after week 4, and the slight increase of the femur’s and scaffold’s BMD overtime. In vivo CT images of the mouse femur and the ohTEBC at weeks 2 (**F**) and 5 (**G**). Blue arrowhead shows the construct, red points at the femur head and green shows the tibia and fibula of the mouse.

To investigate the bone formation in the presence of a humanised haematopoietic system, bone mineralisation was monitored by in vivo CT scans at weeks 2, 3, 5 and 7 following implantation of the ohTEBC (Figure 5A). Both bone volume (BV; mm^3^) and bone mineral density (BMD; mg/cc) were quantified for the scaffold and the femur (Figure S1). We found that the bone mineral density (BMD) and bone volume (BV) of the scaffolds were comparable with previous studies (Figure S1). BV of the scaffold reached stable values after 3–5 weeks, with an average of 20 mm^3^, whereas the BMD slowly increased over time, reaching an average of 600 mg/cc. When comparing the values of BMD (Figure 5C) within the different groups, no substantial differences were seen. Similarly, the comparison of the BV showed no significant differences (Figure 5D), leading us to conclude that the implantation of CD34^+^ cells together with the BM cells did not affect the bone formation rate. Likewise, CT images showed the formation of trabecular bone within the construct (Figure 5F–G), and bone formation in individual mice showed a steady increase in the mouse femur growth and stabilisation of the bone scaffold’s BV. We also did not find any statistical differences between the BV and BMD at week 7 within the different groups (Figure 5B).

### 2.4. Diseased Haematopoiesis in the ohTEBC

**Figure 6:**
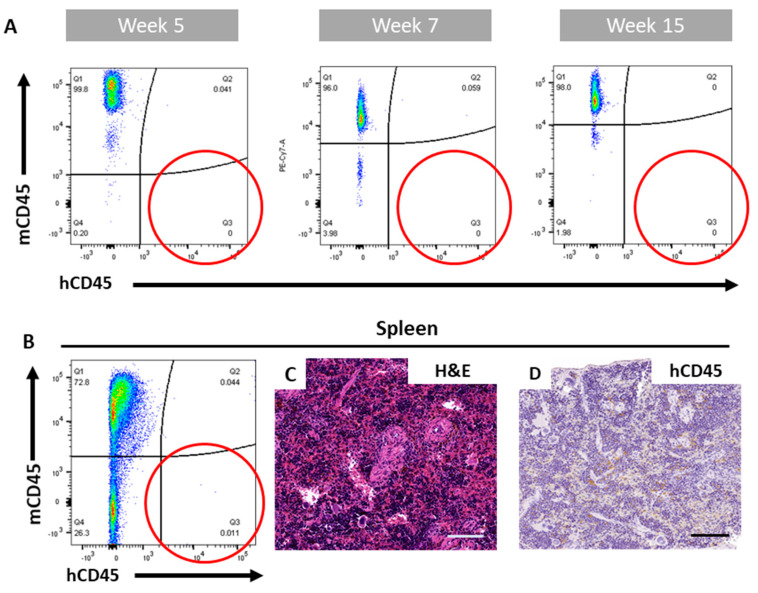
BM-derived CD34^+^ cells from an exemplary multiple myeloma (MM) patient did not engraft when implanting 50,000 cells. Flow cytometry of peripheral blood at weeks 5, 7 and 15 showed no hCD45^+^ circulating cells (**A**). No human cells migrated to the spleen, as demonstrated by flow cytometry analysis (**B**). Histological analysis demonstrated a normal spleen morphology, as shown by H&E (**C**) and no migration of hCD45^+^ cells (**D**). Scale bars: 100 µm.

Next, we wanted to explore the ability of the ohTEBC to sustain a patient-specific and diseased haematopoietic microenvironment, following a similar approach as before. Thus, we implanted BM, as well as CD34^+^ cells, but in this case isolated from the marrow of a patient with MM. A total of 50,000 CD34^+^ cells isolated from three MM patients were engrafted in the ohTEBC, together with 2 × 10^6^ BM cells around the right femur of previously irradiated NSG mice (*n* = 15) (Figure 6). As in previous experiments, the engraftment of CD34^+^ cells was monitored by measuring the presence of hCD45^+^ cells in peripheral blood by flow cytometry.

Measurements were taken at weeks 5 and 7 following engraftment, showing no hCD45^+^ cells in circulation in any of the animals (Figure 6A). To discard the possibility of a delayed engraftment by the CD34^+^ cells derived from the MM patients, an analysis at 15 weeks was performed, which also revealed no presence of hCD45^+^ cells in the peripheral blood (Figure 6A). Flow cytometry analysis of the spleen demonstrated no hCD45^+^ cell migration after 15 weeks (Figure 6B), also corroborated by histological analysis, which revealed a normal spleen morphology with no human cell infiltration (Figure 6C,D). Therefore, it appeared that the implanted CD34^+^ cells from three different MM patients were unable to engraft into NSG mice in this pilot study.

**Figure 7:**
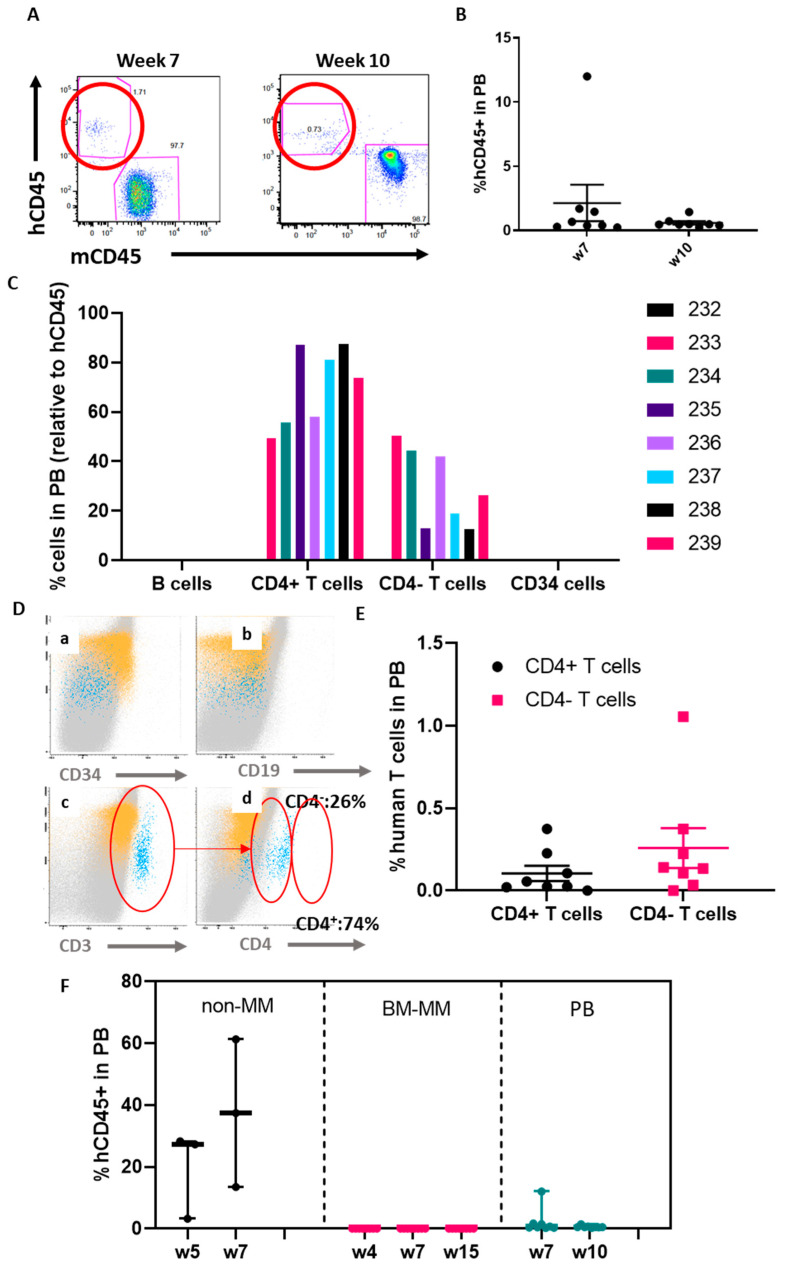
A high dose (120,000 cells) of MM patient-derived CD34^+^ cells is necessary for them to engraft in the construct. In total, 120,000 CD34^+^ cells were implanted in the inner scaffold and FACS was used to monitor its engraftment by detecting hCD45^+^ cells in peripheral blood. At week 7, some engraftment (average of 2%) was detected in peripheral blood (**A**). The engraftment decreased in week 10 in some of the mice (**A**,**B**). Most of the human cells detected were T cells (**C**). Gating strategy for detecting human T cell levels in peripheral blood (**D**). Human T cells were detected in peripheral blood at week 10, with the majority being CD4- T cells (**E**). Comparison of the studies performed with non-MM (85,000 CD34^+^ cells), BM-derived (50,000 CD34^+^ cells) and peripheral blood-derived (120,000 CD34^+^ cells) (**F**). In all graphs the plotted mean with the standard error of the mean (S.E.M.) is depicted.

The lack of engraftment could be related to the abnormal BM microenvironment observed in patients with MM [39,40]. We hypothesised that a higher number of CD34^+^ cells might improve the engraftment (despite increasing the risk of GvHD). As obtaining large numbers of CD34^+^ cells remains a challenge, we utilised CD34^+^ cells obtained from the peripheral blood of a mobilised patient. We were able to collect a much higher number of CD34^+^ cells as compared to isolation from BM aspiration, which allowed us to implant 120,000 CD34^+^ cells per mouse (*n* = 8 mice) (Figure 7). A similar approach was used as in previous experiments, implanting the mononuclear cells, in this case obtained from the peripheral blood, together with the CD34^+^ cells in the construct. Flow cytometry analysis of the peripheral blood seven weeks after the implantation showed some degree of engraftment (Figure 7A). Seven out of the eight implanted mice had around 1%–2% engraftment and one mouse reached a much higher level (12%) (Figure 7B). We then measured hCD45^+^ cells in the peripheral blood at week 10 and the levels decreased as compared to those detected in week 7. Despite low levels, presence of circulating human cells could indicate successful engraftment of the CD34^+^ cells. Furthermore, to investigate the engraftment and survival of the hCD34^+^ cells in the animals, immunohistochemistry (IHC) and flow cytometry of the spleen were conducted, as it is expected for the CD34^+^ cells to migrate to haematopoietic organs such as the spleen. In the case of the mice (Figure S2), hCD34 cells were detected in the spleen by both methods.

Flow cytometry was employed to further characterise the human haematopoietic system 10 weeks after the implantation. Interestingly, no B cell compartment was detected, and most of the human cells detected were T cells (hCD45^+^, hCD19^−^, hCD34^−^, hCD3^−^, hCD4^+^) (Figure 7C). As expected, no circulating hCD34^+^ were detected (Figure 7D). The T cell compartment was reconstituted, differentiating two populations: a CD3^+^/CD4^+^ population and a CD3^+^/CD4^−^ population (Figure 7D,E). In all cases, the CD3^+^/CD4^−^ population was higher.

These results demonstrated that administration of higher doses of CD34^+^ cells may improve hematopoietic cell engraftment, suggesting a correlation between cell dose and human cell engraftment (1%–2% of hCD45^+^ cells in peripheral blood).

## 3. Discussion

In order to study the interaction between the cancer cells and the immune system, an ideal mouse model would need to feature both the tumour and the humanised immune system. Currently, using cord blood-derived CD34^+^ cells is the gold standard for the humanisation of the mouse immune system [13]. A number of successful animal models have been created using this approach, resulting in different degrees of human haematopoiesis in order to study different conditions [41,42,43]. More advanced models of engineering human haematopoiesis in humanised niches have been reported, such as a bioengineered gelatine-based scaffold that has been used to recreate a subcutaneous humanised BM niche, showing chimeric murine-human vasculature [44], or an extramedullary humanised bone that supported both normal and malignant haematopoiesis [45]. Groen et al. reported the generation of a subcutaneous humanised niche in RAG_2_^−/−^γc^−/−^ mice after the implantation of biphasic phosphate calcium particles loaded with mesenchymal stem cells (MSCs). Cord blood-derived CD34^+^ cells and primary MM cells were able to migrate towards this ectopic construct [46]. As mentioned earlier, tissue engineering strategies result in more relevant models, such as the previously mentioned examples, as they also represent a humanised niche supporting stem cell engraftment.

However, despite the high engraftment rates of cord blood-derived CD34^+^ cells and the development of humanised niches, this strategy prevents the modelling of patient-specific haematopoiesis, for which CD34^+^ cells have to be isolated from patient samples, such as the BM or peripheral blood. Werner-Klein et al. studied the engraftment capability of BM-CD34^+^ cells as compared with cord blood derived CD34^+^ after irradiation of NSG mice. The authors concluded that five times more cells are required to achieve similar engraftment levels if the cells are isolated from the BM of carcinoma patients [13].

Here, we demonstrate that the ohTEBC is able to support aged patient-derived CD34^+^ cell engraftment and that the BM niche seems to play a vital role in this process, since the engraftment only occurred with patient-matched BM cells and CD34^+^ cells (Figure 7F). This led to the reconstitution of a humanised haematopoietic system. In particular, human haematopoietic cells were found in the spleen, peripheral blood, humanised BM compartment and in the contralateral leg, achieving levels comparable to other studies using cord blood-derived CD34^+^ cells [47]. These discoveries show promising results towards the development of a patient-specific model of the haematopoietic niche, because CD34^+^ cells can be obtained from a particular patient and engrafted in the same patient-specific niche.

Furthermore, we have proven that the ohTEBC is not only able to engraft CD34^+^ cells, but that a much lower number of CD34^+^ cells is required to achieve similar engraftment levels. We demonstrated that engrafting 85,000 CD34^+^ cells resulted in hCD45^+^ cells in circulation, as compared, for instance, to the 10^6^ CD34^+^ cells per mice used in other successful studies [42]. Due to ethical and logistical constrains, working with patient samples poses a great challenge. Thus, having the potential to reduce the number of cells implanted allows us to first generate a larger animal cohort, which can lead to more significant studies, and, second, removes the requirement of obtaining large patient samples to isolate a sufficient cell number.

To demonstrate the capacity of the ohTEBC to model diseased hematopoiesis, we used BM and CD34^+^ cells obtained from MM patients as examples of diseased marrow. We found that CD34^+^ cells were somehow altered, as a higher number of cells seemed to be a critical factor to detect a low level of human haematopoietic cells in murine tissues as compared to the non-MM patient cells. It had already been described by Gao et al. that the level of CD34^+^ engraftment is dose-dependent, showing that after implantation of 10^4^ cells, between 0%–10% of hCD45 was detected in peripheral blood, and this increased when implanting 10^6^ CD34^+^ cells, achieving engraftments of nearly 80% [48]. In the case of ohTEBC, even after the implantation of a higher number of CD34^+^ cells, the reconstitution levels (hCD45^+^ cells in peripheral blood) were much lower than when using the non-MM derived stem cells (Figure 7F), and the origin of these cells remains unclear, requiring further study. It has been reported that MM cells and HSCs share the same niche and that the MM cells modify this niche, thereby hindering HSC survival [49]. It has also been reported that MM patients present a significant reduction of haematopoietic stem and progenitor cells, as a result of a functional impairment to the HSCs by different mechanisms, including the alteration of the TGFβ pathway [50]. Moreover, when immunodeficient mice received these MM-derived stem cells in the absence of myeloma marrow, normal engraftment levels were achieved [50]. These findings suggest that in our model transplantation of BM from patients may have reproduced the human situation, allowing us to assess the impact of malignant cells on the BM microenvironment. This would be in agreement with results from Brons et al., which demonstrated the importance of the BM microenvironment by transplanting MM-derived HSCs into an immunodeficient mouse [50].

Analysis of the peripheral blood of mice that received CD34^+^ cells from the mobilised patient showed only human T cells (CD4^+^ and CD8^+^). In a study using the Vk*MYC model (noting that this is an immunocompetent strain and a 100% murine model), it was found that mice progressing from smouldering MM (a pre-MM state) to MM presented an elevated number of CD8^+^ T cells [51]. However, this needs to be further studied, as the model used had a murine immune system. In MM patients it has also been reported that clonal cytotoxic T cells are expanded [52]. Our results suggest that having T cells in the ohTEBC model would open the possibility of the use of this model as an immunotherapy drug testing platform, such as for chimeric antigen receptor T (CAR-T) cells. However, this feature will need to be further developed and studied, because the precedence of these human T cells is not completely clear, as they can come from other cell types in the graft, such as long-lived T cells. Moreover, this study is limited, as only one high-dose experiment was performed and more experiments with different CD34^+^ cells will be required to further confirm the results.

In addition to the partially humanised haematopoietic system, the ohTEBC model also features a humanised bone organ and marrow. This is of great importance as it allows us to study the interactions between different cell types residing in this niche. If we take the example of MM, anti-resorptive therapy in MM patients remains a common approach to treat lytic bone pain [53], and numerous novel strategies are being investigated, including epigenetic modification [54,55] and immunomodulatory agents [56]. Here, we have demonstrated that the engraftment of the CD34^+^ cells does not affect the formation of the bone cortical shell, which also opens the door to testing a number of drugs addressing not only bone lesions in bone resorptive states, such as MM, but also potentially bone metastasis in other tumour types.

In conclusion, this model, despite its limitations and early development state, provides a bone compartment and a niche to support the engraftment of CD34^+^ cells from healthy and possibly diseased marrow patients, with the potential to also engraft tumour cells, making it a promising platform in the future of cancer preclinical research.

## 4. Materials and Methods

### 4.1. Patient Sample Processing

BM aspirates from myeloma patients were collected from Greenslopes Private Hospital, Brisbane (GPH). All samples were collected after informed consent and approved by the GPH Research and Ethics Committee (Protocol 17/37) and by the QUT Human Research Ethics Committee (1700000779).

Alternatively, BM and peripheral blood samples from MM patients were collected from Clinica Universitaria de Navarra (CUN) in Pamplona, Spain. Samples were collected after informed consent and approved by the CUN Research Ethics Committee (CEIC 095-2012).

BM aspirates from non-myeloma patients were collected from Holy Spirit Northside Private Hospital. Samples were obtained from patients undergoing knee or hip replacement. All samples were collected after informed consent and approved by the QUT Human Research Ethics Committee (1400001024).

Patients were all female and an average of 70 years old. In the case of MM patients, all were newly diagnosed and had not received any treatment. One of the patients was mobilised after subcutaneous administration of G-CDF (Filgastrin) at 10 µg every 12 h for a total of 5 days. The apheresis was done at day 5.

After the BM aspirate was washed and filtered samples were processed with ACK lysing buffer (A10492, Thermo Fischer Scientific, Waltham, MA, USA), and lysed for 5 min on ice followed by two washes with sterile MACS buffer (130-092-987, Milteny Biotec, Bergisch Gladbach, Germany).

The pellet was then resuspended in MACS buffer and 100 µL of FcR blocking reagent and 100 µL of CD34 Microbeads (130-100-453, Milteny Biotec, Germany) were added for every 10^8^ cells. After incubating for 30 min at 4 °C in the dark, cells were washed once with 10 mL of MACS buffer and taken to the AutoMACS Pro automatic separator (Milteny Biotec, Germany). The program Posseld with double column was used for the separation. Once the positive fraction was isolated, the purity of the cells was verified by CD34^+^ flow cytometry, obtaining >95% purity from the BM aspirate samples and a lower 65.57% in the peripheral blood from the mobilised patient. The cells were kept frozen in 90% heat-inactivated fetal bovine serum (FBS) and 10% DMSO [57].

### 4.2. ohTEBC Generation and Characterisation

Medical grade polycaprolactone (mPCL) tubular scaffolds were fabricated using MEW technology, printed with a made-in-house device, as previously described [19]. Briefly, the mPCL was heated above its melting point and extruded through a 23 G needle [27] and deposited onto a rotating grounded mandrill of 3 mm diameter for the BM scaffold and 4 mm diameter for the bone (outer) scaffold. Both scaffolds had a length of 6 mm. A high voltage was applied to the polymer jet in order to control and reduce the fibre diameter, achieving a stable fibre jet throughout the printing process. The controlled fibre diameter was set at 8 µm and the pore size at 150 µm.

Primary hOBs were isolated from patients undergoing hip or knee replacement surgery after informed consent (QUT HREC approval no. 1400001024) as previously described [28]. The scaffolds were coated with a layer of CaP [58] and placed vertically on a sterile 6-well plate. A total of 4 × 10^5^ hOBs in 50 µL were seeded onto the scaffolds and allowed to attach for 4 h at 37 °C in the incubator. To prevent the seeded scaffolds from evaporating, 10 µL of serum-free medium was added every 30 min. Then, 2 mL of basal medium (alpha-MEM, 10% FBS 100 IU/mL penicillin and 100 μg/mL streptomycin, all Thermo Fischer Scientific) was added into the 6-well plate. 

Scaffolds were maintained in basal medium in static culture conditions for 2 weeks, monitoring the hOB viability and expansion using a live/dead assay. Briefly, 1 µL of fluorescein diacetate (FDA; 10 mg/mL, Invitrogen, Carlsbad, CA, USA) was added, together with 1 µL of propidium iodide (PI; 5 mg/mL, Invitrogen, CA, USA) in 1 mL of sterile phosphate buffered saline (PBS) (Thermo Fischer Scientific). Previously PBS-washed scaffolds were incubated in the FDA/PI mixture at room temperature (RT) for 5 min, followed by three 5 min washes with PBS. Scaffolds were then transferred to a glass slide and imaged using a confocal microscope (Leica Sp5 confocal, Leica, Wetzlar, Germany) using a 2 µm step size. Results were analysed as a maximum projection of the Z stack of 200 µm. Confocal images were taken fortnightly. When cells were confluent on the scaffold fibres (2 weeks), the cultures were supplemented with 10 mM β-glycerophosphate, 0.17 mM ascorbic acid and 100 nM dexamethasone (all Sigma-Aldrich, St. Louis, MO, USA). The osteogenic medium stimulated the ECM mineralisation deposition [59]. The scaffolds with the hOBs were kept in the osteogenic medium for 6–8 weeks before in vivo implantation.

### 4.3. In Vivo Implantation

6–8-week-old NOD/SCID IL2Rγ^null^ (NSG) mice were purchased from the Animal Resources Centre (Canning Vale, Australia) and acclimatised in a specific pathogen-free, temperature and light-controlled environment. Animal ethics were obtained from the University of Queensland QUT/279/17 and were subsequently approved by administrate review by the QUT University Animal Ethics Committee (1700000935). For the experiments performed at the Centre for Applied Medical Research in Pamplona, Spain, animal ethics were also obtained by the Ethics Committee for Animal Experiments (CEEA) from the University of Navarra (112-18). All animal experiments were conducted in accordance with the Australian Code of Practice for the Care and Use of Animals for Scientific Purposes. Animals had sterilised water and food ad libitum for the duration of the experiment. Mice were gamma irradiated with a dose of 1.8 Gy 12–16 h before the surgery. The animals were anaesthetised via intraperitoneal injection of ketamine (10 mg/mL) and xylazine (1 mg/mL) in sterile saline solution at a dosage of 10 µL/g of body weight. Mice were then transferred to a separate cage until they became unconscious and ready for surgery. Eye ointment was applied to protect eyes during the surgical procedure.

The right leg of the mouse was shaved and sterilised with 70% ethanol or chlorhexidine gluconate. A longitudinal, lateral skin incision along the femur (10–15 mm) was performed, using a sterile scalpel, from the hip joint to the knee. With a pair of sterile blunt-end tweezers, the femur was then exposed. A longitudinal incision of the fascia lata followed. M. vastus lateralis and M. biceps femoris were split by blunt dissection and M. tensor fasciae latae was lifted to the distal part/methaphysis of the femur (preserving the sciatic nerve). The integrity of the femoral nerve or any major artery was maintained, as damage to these parts may result in hind limb paralysis. Using a 27G (insulin) syringe, a 0.5 mm cortical window at the anterior femur was made by drilling into the bone cortex to create a communication with the mouse BM and the implanted human BM cells.

The ohTEBC was created by combining an inner and outer scaffold. The inner scaffold consisted of a 5-mm-long and 3-mm-diameter mPCL scaffold containing 1–2 × 10^6^ human BM cells from 70-year-old patients isolated from BM aspirates in 35 µL of fibrin glue (TISSEEL, Baxter, USA), and was wrapped around the diaphysis of the femur. The bone scaffold was then implanted around the inner scaffold. To be able to wrap the outer scaffold (containing the hOBs) around the inner scaffold (containing the BM cells and the CD34^+^ cells) a longitudinal cut using a pair of sterile scissors was done. Twenty micrograms of clinical rhBMP-2 (Medtronic, Ireland), together with 20 µL of fibrin glue, was added to the interface of both scaffolds to stimulate bone formation and to also secure both scaffolds together.

The divided muscles were sutured using resorbable sutures and the skin closed with autoclips. Animals were given buprenorphine every 6 h (0.1 mg/kg) for the 72 h after the surgery and wounds as well as limb mobility were closely monitored.

### 4.4. Bone Formation Monitoring

The humanised bone was allowed to develop in vivo for 4–8 weeks, monitored by in vivo µ-computed tomography (µCT). Animals were anaesthetised with 2% isoflurane, 1 L O_2_/min and scanned in an Inveon Micro-CT/PET (positron emission tomography) Image Station (Inveon, Siemens, Munich, Germany). Mice were scanned at 80 KV and 500 µA, with an effective pixel size of 35.84 µm and an exposure time of 1100 ms with a binning factor of 2. A 0.5 mm aluminium filter was used with 180 projections at 360° rotations at medium system magnification (source-to-centre = 184.24 mm, source-to-detector = 345.34 mm). These settings were maintained throughout the experiment, and a calibrated phantom (Siemens, Germany) was scanned to calculate BMD values expressed as mg/cc.

Results from the PET-CT scans were analysed using the Siemens Inveon software; noise and ring artefact reduction were applied. A lower threshold of 500 HU was applied for every image, and two regions of interest (ROIs) were used, one for the femur and one for the scaffold. Total BV (mm^3^) and the Hounsfield units (HU) for each of the ROIs were obtained for each sample.

### 4.5. Histology and Immunohistochemistry (IHC)

Murine tissues were fixed at 4 °C in 4% paraformaldehyde (PFA) for 24 h before transfer to and storage in 70% ethanol at 4 °C. Bone tissues were decalcified in a rapid decalcifier (microwave histoSTATION, Milestone Medical, Australia) containing 10% EDTA, pH 7.4 solution at 37 °C with gentle agitation. To verify if the decalcification process was successful (2 weeks for mouse femurs), a scout view with the µCT-40 scanner (Scanco Medical) was performed, to confirm that there was no calcified tissue left.

Fixed tissues were dehydrated overnight in a tissue processor (ExcelsiorES, Thermo Fischer Scientific, USA), embedded in paraffin and cut into 5-µm sections on poly-L-lysine coated slides. The sections were then transferred to a dehydration oven for a minimum of 4 h to fix the tissue to the slides. After deparaffinisation, slides were washed in DAKO wash buffer (DAKO, Santa Clara, CA, USA) before performing antigen retrieval. The slides were heat-treated at 95 °C for 5 min in sodium citrate buffer (10 mM Sodium Citrate, 0.05% Tween-20, pH 6.0) or Tris-EDTA buffer (10 mM Tris Base, 1 mM EDTA, 0.05% Tween-20, pH 9.0) in a Decloaking Chamber (Biocare Medical, Concord, CA, USA) or incubated for 15 min in Proteinase K solution (DAKO, USA) at room temperature (RT), to retrieve the antigen. After two more washes, slides were incubated with 3% H_2_O_2_ (Sigma-Aldrich) to block endogenous peroxidase activity for 5 min (10 min for spleen sections). Staining for intracellular proteins involved an extra step of permeabilisation with 0.1% Triton X-100 (Merck, Kenilworth, NJ, USA) in PBS. After three washes, the slides were blocked for 30 min with a 2% BSA (Sigma-Aldrich) in PBS solution before adding the primary antibody (the concentrations and incubation time differed varied depending on the antibody used (Table S1). For signal detection, the Envision+Dual Link secondary HRP system (Dako) and 3,3′-DAB chromogen substrate (Dako) was used. Samples were then counterstained with haematoxylin (Sigma-Aldrich) to visualise cell nuclei, before dehydration and mounting.

### 4.6. Statistical Analysis

In all cases data were analysed using GraphPad (8.2.1) or R (3.5). Lines in boxplots and whisker plots indicate the median, top and bottom lines corresponding to the 25% and 75% percentiles. The dots indicate outliers after ANOVA test. In all cases significance was considered to be *p* ≤ 0.05.

### 4.7. Flow Cytometry

Peripheral blood (200 µL total) was collected by retro-orbital bleeding fortnightly, alternating the right and the left eye. Thirty microlitres were transferred to an 0.6 mL Eppendorf tube and allowed to clot at RT for 30 min–2 h, after which the blood was centrifuged at 1000× *g* for 10 min. The serum was then carefully collected and stored at −80 °C until further analysis.

The remaining 170 µL of peripheral blood was mixed with 6 µL of 200 mM EDTA and kept at 4 °C. The red blood cells (RBC) were lysed by adding 2 mL of ACK lysing buffer for 10 min at 4 °C. To stop the lysis reaction, 10 mL of sterile PBS was added and centrifuged at 350× *g* for 10 min. The pellet was resuspended in MACS buffer for antibody staining for 30 min at 4 °C. The cells were then washed once with MACS buffer and analysed using flow cytometry (Fortessa X20, BD Biosciences, San Jose, CA, USA).

For the flow cytometry analysis, unstained samples, single colour controls (SCCs) and fluorescence minus one (FMO) were used for all of the experiments using pooled blood from the animals. All samples were stained for 30 min at 4 °C in the dark and washed with MACS buffer once. A BD LSR Fortessa X20 Flow cytometer with a 355 nm, 405 nm, 488 nm, 561 nm and a 633 nm laser setup was used for all of the experiments. To analyse the results, the software Flowjo_v10 was used. Channel compensation was routinely performed in all of the experiments, and the populations were gated according to the FMO and SCCs. hCD45 antibody was routinely used to monitor the engraftment of the patient-derived CD34^+^ cells (Table S2).

## 5. Conclusions

Humanised mice with tissue-engineered constructs provide a unique opportunity to develop models that better mimic human disease, allowing for the study of BM microenvironment interactions and enabling drug-testing studies, facilitating translatable research outcomes. The studies depicted in this manuscript provide the first steps towards the development of a patient-specific platform for preclinical research, especially suited for studying the immune system and the BM microenvironment. A genuinely representative animal model, especially for cancer studies, should have a humanised haematopoietic system, together with a humanised microenvironment. The initial results presented here, although promising, must be taken in perspective, as a relatively low number of mice per group was employed. Future experiments with a larger number of patients and different CD34^+^ cell doses will be required to confirm these findings. Here, we have shown that the ohTEBC is able to support human haematopoiesis more efficiently than previously reported studies which do not employ an ohTEBC, as less cells are required to achieve engraftment, which greatly facilitates the development of the model. Moreover, the new bone formation was not affected by the presence of human haematopoiesis, which makes this model suitable to study bone-related conditions, such as myeloma bone disease or bone metastasis.

The ohTEBC also created a diseased microenvironment, using MM samples as an example of a diseased marrow. One of the major barriers in this model is obtaining a sufficient amount of CD34^+^ cells from a marrow aspirate, which is especially problematic in conditions affecting the BM. MM CD34^+^ cells showed much lower engraftment potential than cells derived from healthy marrow, and an even higher number of cells (120,000) was required to show discrete engraftment levels. However, we demonstrated that these cells can be isolated and enriched from peripheral blood, when having access to larger cell numbers. This could mean that in only a few cases a patient-specific model could be achieved, as having access to mobilised patient samples is rare. However, the possibility of having a humanised haematopoietic system in a mouse model opens the door for a plethora of applications, including drug testing and the study of interactions within the microenvironment.

In conclusion, we have demonstrated very promising results using the ohTEBC as a platform to model a patient-specific human haematopoietic niche, which also possibly supports haematopoiesis in haematological malignancies. We have also demonstrated that the engraftment depends on the patient status, and that diseased marrows can contain CD34^+^ cells with less engraftment potential. Furthermore, we have shown that peripheral blood can be used as a source of CD34^+^ cells in mobilised patients. Broader development of the model is required to study in greater depth the healthy and diseased haematopoietic niches, followed by the engraftment of tumour cells. We believe that in the future, models such as this one will facilitate a bench-to-bedside transition of more safe and efficacious novel therapeutic strategies, bridging the gap between drug discovery and development to clinical trials, thus translating more safe and efficacious drugs into clinical practice.

## Supplementary Materials

The following are available online at https://www.mdpi.com/2072-6694/12/8/2205/s1, Figure S1: Analysis procedure of the in vivo CT scans and average values of bone formation. BV of the right femur (Green region of interest (ROI)) and scaffold (Pink ROI), visualised with the Siemens Inveon software (A). Regression line to calculate BMD values from BV using a calibrated phantom (B). Box plot showing the combined values of the scaffold BV (C) of all patients implanted in the ohTEBC (*n* = 41 mice), Figure S2: IHC showed hCD34 cells in the spleen. Results were confirmed via flow cytometry; % of hCD34 cells detected shown as % of hCD45 cells, Table S1: Details of the antibodies used for IHC, item number and details for the IHC staining protocol; working dilution, incubation time and antigen retrieval method, Table S2: Conjugated antibodies used in flow cytometry experiments. All antibodies were used in the concentrations advised by the manufacturer.

## References

[1] Perrin S. (2014). Make mouse studies work. Nature.

[2] Harrison R.K. (2016). Reason for failure in phase II d Reason for failure in phase III Phase II and phase III failures: 2013–2015. Nat. Rev. Drug Discov..

[3] Thibaudeau L., Quent V.M., Holzapfel B.M., Taubenberger A.V., Straub M., Hutmacher D.W. (2014). Mimicking breast cancer-induced bone metastasis in vivo: Current transplantation models and advanced humanized strategies. Cancer Metastasis Rev..

[4] Holzapfel B.M., Thibaudeau L., Hesami P., Taubenberger A., Holzapfel N.P., Mayer-Wagner S., Power C., Clements J., Russell P., Hutmacher D.W. (2013). Humanised xenograft models of bone metastasis revisited: Novel insights into species-specific mechanisms of cancer cell osteotropism. Cancer Metastasis Rev..

[5] Zschaler J., Schlorke D., Arnhold J. (2014). Differences in innate immune response between man and mouse. Crit. Rev. Immunol..

[6] Gonzalez H., Hagerling C., Werb Z. (2018). Roles of the immune system in cancer: From tumor initiation to metastatic progression. Genes Dev..

[7] Walsh N.C., Kenney L.L., Jangalwe S., Aryee K.-E., Greiner D.L., Brehm M.A., Shultz L.D. (2017). Humanized Mouse Models of Clinical Disease. Annu. Rev. Pathol. Mech. Dis.

[8] Carrillo M.A., Zhen A., Kitchen S.G. (2018). The use of the humanized mouse model in gene therapy and immunotherapy for HIV and cancer. Front. Immunol..

[9] McCune J.M., Namikawa R., Kaneshima H., Shultz L.D., Lieberman M., Weissman I.L. (1988). The SCID-hu mouse: Murine model for the analysis of human hematolymphoid differentiation and function. Science (80-.).

[10] Lapidot T., Pflumio F., Doedens M., Murdoch B., Williams D.E., Dick J.E. (1992). Cytokine stimulation of multilineage hematopoiesis from immature human cells engrafted in SCID Mice. Science (80-.).

[11] Brehm M., Shultz L., Greiner D. (2010). Humanized Mouse Models to Study Human Diseases. Curr. Opin. Endocrinol. Diabetes Obes..

[12] Holzapfel B.M., Wagner F., Thibaudeau L., Levesque J.P., Hutmacher D.W. (2015). Concise review: Humanized models of tumor immunology in the 21st century: Convergence of cancer research and tissue engineering. Stem Cells.

[13] Werner-Klein M., Proske J., Werno C., Schneider K., Hofmann H.S.H.-S., Rack B., Buchholz S., Ganzer R., Blana A., Seelbach-Göbel B. (2014). Immune humanization of immunodeficient mice using diagnostic bone marrow aspirates from carcinoma patients. PLoS ONE.

[14] King M.A., Covassin L., Brehm M.A., Racki W., Pearson T., Leif J., Laning J., Fodor W., Foreman O., Burzenski L. (2009). Human peripheral blood leucocyte non-obese diabetic-severe combined immunodeficiency interleukin-2 receptor gamma chain gene mouse model of xenogeneic graft-versus-host-like disease and the role of host major histocompatibility complex. Clin. Exp. Immunol..

[15] Plaks V., Kong N., Werb Z. (2015). The cancer stem cell niche: How essential is the niche in regulating stemness of tumor cells?. Cell Stem Cell.

[16] Calimeri T., Battista E., Conforti F., Neri P., Di Martino M.T., Rossi M., Foresta U., Piro E., Ferrara F., Amorosi A. (2011). A unique three-dimensional SCID-polymeric scaffold (SCID-synth-hu) model for in vivo expansion of human primary multiple myeloma cells. Leukemia.

[17] Wagner F., Holzapfel B.M., Thibaudeau L., Straub M., Ling M.T., Grifka J., Loessner D., Levesque J.P., Hutmacher D.W. (2016). A Validated Preclinical Animal Model for Primary Bone Tumor Research. J. Bone Jt. Surg. Am. Vol..

[18] Landgraf M., Lahr C.A., Sanchez-Herrero A., Meinert C., Shokoohmand A., Pollock P.M., Hutmacher D.W., Shafiee A., McGovern J.A. (2019). Humanized bone facilitates prostate cancer metastasis and recapitulates therapeutic effects of zoledronic acid in vivo. Bone Res..

[19] Martine L.C., Holzapfel B.M., Mcgovern J.A., Quent V.M., Hesami P., Wunner F.M., De-juan-pardo E.M., Brown T.D., Nowlan B., Jing D. (2017). Engineering a humanized bone organ in mice to study bone metastases. Nat. Protoc..

[20] Fulciniti M., Tassone P., Hideshima T., Vallet S., Nanjappa P., Ettenberg S.A., Shen Z., Patel N., Tai Y.T., Chauhan D. (2009). Anti-DKK1 mAb (BHQ880) as a potential therapeutic agent for multiple myeloma. Blood.

[21] Hameed A., Brady J.J., Dowling P., Clynes M., O’Gorman P. (2014). Bone disease in multiple myeloma: Pathophysiology and management. Cancer Growth Metastasis.

[22] Hu J., Handisides D.R., Van Valckenborgh E., De Raeve H., Menu E., Vande Broek I., Liu Q., Sun J.D., Van Camp B., Hart C.P. (2010). Targeting the multiple myeloma hypoxic niche with TH-302, a hypoxia-activated prodrug. Blood.

[23] Landgraf M., McGovern J.A., Friedl P., Hutmacher D.W. (2018). Rational Design of Mouse Models for Cancer Research. Trends Biotechnol..

[24] Holzapfel B.M., Hutmacher D.W., Nowlan B., Barbier V., Thibaudeau L., Theodoropoulos C., Hooper J.D., Loessner D., Clements J.A., Russell P.J. (2015). Tissue engineered humanized bone supports human hematopoiesis in vivo. Biomaterials.

[25] Thibaudeau L., Taubenberger A.V., Holzapfel B.M., Quent V.M., Fuehrmann T., Hesami P., Brown T.D., Dalton P.D., Power C.A., Hollier B.G. (2014). A tissue-engineered humanized xenograft model of human breast cancer metastasis to bone. Dis. Model. Mech..

[26] Baldwin J.G., Wagner F., Martine L.C., Holzapfel B.M., Theodoropoulos C., Bas O., Savi F.M., Werner C., De-Juan-Pardo E.M., Hutmacher D.W. (2017). Periosteum tissue engineering in an orthotopic in vivo platform. Biomaterials.

[27] Brown T.D., Slotosch A., Thibaudeau L., Taubenberger A., Loessner D., Vaquette C., Dalton P.D., Hutmacher D.W. (2012). Design and fabrication of tubular scaffolds via direct writing in a melt electrospinning mode. Biointerphases.

[28] Reichert J.C., Quent V.M.C., Burke L.J., Stansfield S.H., Clements J.A., Hutmacher D.W. (2010). Mineralized human primary osteoblast matrices as a model system to analyse interactions of prostate cancer cells with the bone microenvironment. Biomaterials.

[29] Li Z., Hardij J., Bagchi D.P., Scheller E.L., MacDougald O.A. (2018). Development, regulation, metabolism and function of bone marrow adipose tissues. Bone.

[30] Liu H., He J., Koh S.P., Zhong Y., Liu Z., Wang Z., Zhang Y., Li Z., Tam B.T., Lin P. (2019). Reprogrammed marrow adipocytes contribute to myeloma-induced bone disease. Sci. Transl. Med..

[31] Allegra A., Innao V., Gerace D., Allegra A.G., Vaddinelli D., Bianco O., Musolino C. (2018). The adipose organ and multiple myeloma: Impact of adipokines on tumor growth and potential sites for therapeutic intervention. Eur. J. Intern. Med..

[32] Zweegman S., Engelhardt M., Larocca A. (2017). Elderly patients with multiple myeloma: Towards a frailty approach?. Curr. Opin. Oncol..

[33] Winters S., Martin C., Murphy D., Shokar N.K. (2017). Breast Cancer Epidemiology, Prevention, and Screening. Progress in Molecular Biology and Translational Science.

[34] Hofgaard P.O., Jodal H.C., Bommert K., Huard B., Caers J., Carlsen H., Schwarzer R., Schünemann N., Jundt F., Lindeberg M.M. (2012). A Novel Mouse Model for Multiple Myeloma (MOPC315.BM) That Allows Noninvasive Spatiotemporal Detection of Osteolytic Disease. PLoS ONE.

[35] Ali N., Flutter B., Rodriguez R.S., Sharif-Paghaleh E., Barber L.D., Lombardi G., Nestle F.O. (2012). Xenogeneic Graft-versus-Host-Disease in NOD-scid IL-2Rc null Mice Display a T-Effector Memory Phenotype. PLoS ONE.

[36] Macedo F., Ladeira K., Pinho F., Saraiva N., Bonito N., Pinto L., Gonçalves F. (2017). Bone metastases: An overview. Oncol. Rev..

[37] Reagan M.R., Rosen C.J. (2016). Navigating the bone marrow niche: Translational insights and cancer-driven dysfunction. Nat. Rev. Rheumatol..

[38] Kovacic N., Croucher P.I., McDonald M.M. (2014). Signaling between tumor cells and the host bone marrow microenvironment. Calcif. Tissue Int..

[39] Noonan K., Borrello I. (2011). The immune microenvironment of myeloma. Cancer Microenviron..

[40] Balakumaran A., Robey P.G., Fedarko N., Landgren O. (2010). Bone marrow microenvironment in myelomagenesis: Its potential role in early diagnosis. Expert Rev. Mol. Diagn..

[41] Minoda Y., Virshup I., Rojas I.L., Haigh O., Wong Y., Miles J.J., Wells C.A., Radford K.J. (2017). Human CD141+ dendritic cell and CD1c+ dendritic cell undergo concordant early genetic programming after activation in humanized mice in vivo. Front. Immunol..

[42] Haworth K.G., Ironside C., Norgaard Z.K., Obenza W.M., Adair J.E., Kiem H.P. (2017). In Vivo Murine-Matured Human CD3+Cells as a Preclinical Model for T Cell-Based Immunotherapies. Mol. Ther. Methods Clin. Dev..

[43] Abarrategi A., Foster K., Hamilton A., Mian S.A., Passaro D., Gribben J., Mufti G., Bonnet D. (2017). Versatile humanized niche model enables study of normal and malignant human hematopoiesis. J. Clin. Investig..

[44] Passaro D., Abarrategi A., Foster K., Ariza-McNaughton L., Bonnet D. (2017). Bioengineering of humanized bone marrow microenvironments in mouse and their visualization by live imaging. J. Vis. Exp..

[45] Chen Y., Jacamo R., Shi Y.X., Wang R.Y., Battula V.L., Konoplev S., Strunk D., Hofmann N.A., Reinisch A., Konopleva M. (2012). Human extramedullary bone marrow in mice: A novel in vivo model of genetically controlled hematopoietic microenvironment. Blood.

[46] Groen R.W.J., Noort W.A., Raymakers R.A., Prins H.J., Aalders L., Hofhuis F.M., Moerer P., Van Velzen J.F., Bloem A.C., Van Kessel B. (2012). Reconstructing the human hematopoietic niche in immunodeficient mice: Opportunities for studying primary multiple myeloma. Blood.

[47] Ishikawa F., Yasukawa M., Lyons B., Yoshida S., Miyamoto T., Yoshimoto G., Watanabe T., Akashi K., Shultz L.D., Harada M. (2005). Development of functional human blood and immune systems in NOD/SCID/IL2 receptor γ chain null mice. Blood.

[48] Gao Z., Fackler M.J., Leung W., Lumkul R., Ramirez M., Theobald N., Malech H.L., Civin C.I. (2001). Human CD34+ cell preparations contain over 100-fold greater NOD/SCID mouse engrafting capacity than do CD34- cell preparations. Proc. Exp. Hematol..

[49] Noll J.E., Williams S.A., Purton L.E., Zannettino A.C.W. (2012). Tug of war in the haematopoietic stem cell niche: Do myeloma plasma cells compete for the HSC niche?. Blood Cancer J..

[50] Bruns I., Cadeddu R.P., Brueckmann I., Fröbel J., Geyh S., Büst S., Fischer J.C., Roels F., Wilk C.M., Schildberg F.A. (2012). Multiple myeloma-related deregulation of bone marrow-derived CD34 + hematopoietic stem and progenitor cells. Blood.

[51] Calcinotto A., Ponzoni M., Ria R., Grioni M., Cattaneo E., Villa I., Teresa M., Bertilaccio S., Chesi M., Rubinacci A. (2015). Modifications of the mouse bone marrow microenvironment favor angiogenesis and correlate with disease progression from asymptomatic to symptomatic multiple myeloma. Oncoimmunology.

[52] Sze D.M.Y., Giesajtis G., Brown R.D., Raitakari M., Gibson J., Ho J., Baxter A.G., De Groth B.F.S., Basten A., Joshua D.E. (2001). Clonal cytotoxic T cells are expanded in myeloma and reside in the CD8+CD57+CD28- compartment. Blood.

[53] Baron R., Ferrari S., Graham R., Russell G. (2011). Denosumab and bisphosphonates: Different mechanisms of action and effects. Bone.

[54] Dimopoulos K., Gimsing P., Grønbæk K. (2014). The role of epigenetics in the biology of multiple myeloma. Blood Cancer J..

[55] Canella A., Nieves H.C., Sborov D.W., Cascione L., Radomska H.S., Smith E., Stiff A., Consiglio J., Caserta E., Rizzotto L. (2015). HDAC inhibitor AR-42 decreases CD44 expression and sensitizes myeloma cells to lenalidomide. Oncotarget.

[56] Sondergeld P., van de Donk N.W.C.J., Richardson P.G., Plesner T. (2015). Monoclonal antibodies in myeloma. Clin. Adv. Hematol. Oncol..

[57] Berz D., McCormack E.M., Winer E.S., Colvin G.A., Quesenberry P.J. (2007). Cryopreservation of hematopoietic stem cells. Am. J. Hematol..

[58] Vaquette C., Ivanovski S., Hamlet S.M., Hutmacher D.W. (2013). Effect of culture conditions and calcium phosphate coating on ectopic bone formation. Biomaterials.

[59] Muerza-Cascante M.L., Shokoohmand A., Khosrotehrani K., Haylock D., Dalton P.D., Hutmacher D.W., Loessner D. (2016). Endosteal-like extracellular matrix expression on melt electrospun written scaffolds. Acta Biomater..

