# Supplementary material for: Engineering a Humanised Niche to Support Human Haematopoiesis in Mice: Novel Opportunities in Modelling Cancer
Source: Cancers (Basel). 2020 Aug 6;12(8):2205. doi: 10.3390/cancers12082205 (PMC7466161; doi:10.3390/cancers12082205)
Supplement: Supplementary file 1 [file cancers-12-02205-s001.pdf]

Article

Engineering a Humanised Niche to Support Human Haematopoiesis in Mice: Novel Opportunities in Modelling Cancer

Supplementary

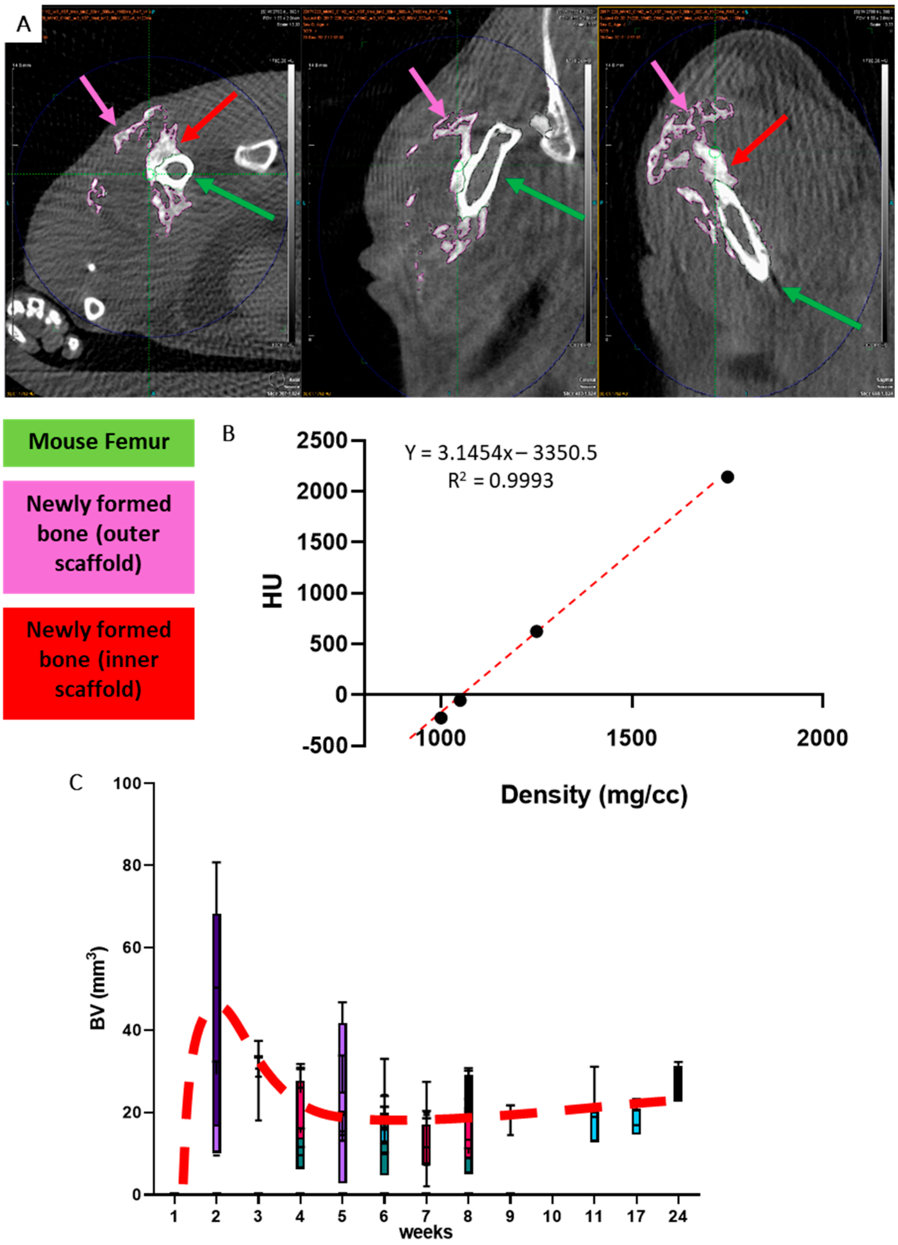

Figure S1. Analysis procedure of the *in vivo* CT scans and average values of bone formation. BV of the right femur (Green ROI) and scaffold (Pink ROI). Visualised with the Siemens Inveon Software [A]. Regression line to calculate BMD values from BV using a calibrated phantom [B]. Box plot showing the combined values of the scaffolds BV [C] of all patients implanted in the ohTEBC (n=41 mice).

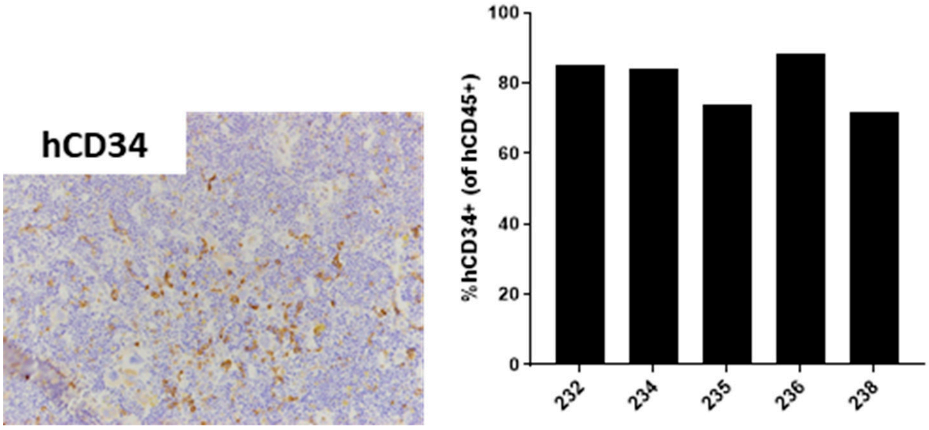

Figure S2. IHC showed hCD34 cells in the Spleen. Results were confirmed via Flow cytometry % of hCD34 cells detected shown as % of hCD45 cells.

Table S1. Details of the antibodies used for IHC, item number, and details for the IHC staining protocol; Working dilution, Incubation time and antigen retrieval method.

Antibody	Product #	Species	Working Dilution	Incubation	Antigen Retrieval
**hs-Col-1**	Abcam (ab23446)	Mouse	1:100	o/n @ 4°C	Proteinase K 15 min/RT
**Lamin A/C**	Abcam (ab108595)	Rabbit	1:300	1 hr @ RT	EDTA Buffer, pH 9.0, 95°C/ 5 min
**NuMA**	Abcam (ab97585)	Rabbit	1:200	1 hr @ RT	Sodium Citrate pH 6.0, 95°C/ 5 min
**hCD45**	Dako (M0701)	Rabbit	1:300	1 hr @ RT	Sodium Citrate pH 6.0, 95°C/ 5 min

Table S2. Conjugated antibodies used in flow cytometry experiments. All antibodies were used in the concentrations advised by the manufacturer.

	Fluorophore	Clone	REF
**mCD45**	V500	30-F11	BD-553081
**mCD45**	PE-Cy7	30-F11	BD-552849
**hCD45**	Pacific Orange	HI30	BD-560777
**hCD45**	APC	HI30	BD-555485
**hCD34**	PerCP-Cy5.5	8G12	BD 347222
**hCD33**	APC	P67.6	BD 340474
**hCD19**	PE-Cy7	J3-119	Beckman Coulter IM3628
**hCD3**	APC-H7	SK7	BD-641415
**hCD4**	Pacific Blue	RPA-T4	BioLegend-300527
